# Using image analysis for quantitative assessment of needle bladder rust disease of Norway spruce

**DOI:** 10.1111/ppa.12842

**Published:** 2018-03-01

**Authors:** A. Ganthaler, A. Losso, S. Mayr

**Affiliations:** ^1^ Department of Botany University Innsbruck Sternwartestrasse 15 Innsbruck A‐6020 Austria

**Keywords:** fungal pathogen, phenotyping, *Picea abies*, plant disease, subalpine, unmanned aerial vehicle (UAV)

## Abstract

High elevation spruce forests of the European Alps are frequently infected by the needle rust *Chrysomyxa rhododendri*, a pathogen causing remarkable defoliation, reduced tree growth and limited rejuvenation. Exact quantification of the disease severity on different spatial scales is crucial for monitoring, management and resistance breeding activities. Based on the distinct yellow discolouration of attacked needles, it was investigated whether image analysis of digital photographs can be used to quantify disease severity and to improve phenotyping compared to conventional assessment in terms of time, effort and application range. The developed protocol for preprocessing and analysis of digital RGB images enabled identification of disease symptoms and healthy needle areas on images obtained in ground surveys (total number of analysed images *n *=* *62) and by the use of a semiprofessional quadcopter (*n *=* *13). Obtained disease severities correlated linearly with results obtained by manual counting of healthy and diseased needles for all approaches, including images of individual branches with natural background (*R* ^2^ = 0.87) and with black background (*R* ^2^ = 0.95), juvenile plants (*R* ^2^ = 0.94), and top views and side views of entire tree crowns of adult trees (*R* ^2^ = 0.98 and 0.88, respectively). Results underline that a well‐defined signal related to needle bladder rust symptoms of Norway spruce can be extracted from images recorded by standard digital cameras and using drones. The presented protocol enables precise and time‐efficient quantification of disease symptoms caused by *C. rhododendri* and provides several advantages compared to conventional assessment by manual counting or visual estimations.

## Introduction

Climate change is expected to drive changes in the dynamics of forest insect and pathogen attacks (Ayres & Lombardero, 2000; Allen *et al*., 2010), demanding innovative approaches and the application of new technologies in monitoring of forest health. In subalpine Norway spruce forests of the European Alps, the needle bladder rust (*Chrysomyxa rhododendri*) is one of the most frequent forest pathogens. For example, more than 20 000 hectares of infected spruce forest have been reported in the alpine region of Tyrol, Austria during the last decades (Bauer & Schwaninger, 2007; Ganthaler *et al*., 2014). The rust fungus undergoes a complex life cycle with different spore types and a host shift between the telial host *Rhododendron* sp. and the aecial host *Picea abies* (Gäumann, 1946; Crane, 2001). Thus, infections are frequent on subalpine forest sites where both hosts co‐occur. The pathogen overwinters in the rhododendron leaves and forms basidiospores on the lower leaves in spring. Spores are spread by the wind and infect the fresh current‐year spruce needles. Within a few weeks, the pathogen forms an extensive mycelium within the needles, causing chlorophyll degradation and distinct yellowing of infected needle parts. During August, aeciospores are released and again infect rhododendron plants, while spruce needles with disease symptoms are shed by the tree at the end of summer. Infections by *C. rhododendri* can cause the loss of up to 90% of the current‐year foliage, as reported for subalpine spruce forest in Tyrol, Austria for the years 2011, 2012 and 2015 (Ganthaler *et al*., 2017b). Repeatedly attacked trees show several distinct anatomical, morphological and physiological modifications, such as reduced chlorophyll content and reduced net photosynthesis of the needles, diminished biomass accumulation by all plant organs, and lower annual ring width and height increment (Ganthaler *et al*., 2014). The lower growth rate causes reduced timber yield and severe problems with rejuvenation of subalpine spruce forests (Oberhuber *et al*., 1999; Plattner *et al*., 1999; Bauer *et al*., 2000). Therefore, both negative economic effects and negative impact on protection forests arise in the Central European Alps, where Norway spruce is one of the most frequent conifer species.

For the appraisement of damage, the implementation of management actions and scientific analyses, a precise and time‐efficient determination of disease severity on different spatial scales is essential. Current approaches are either not completely reliable or require substantial investment in time and labour, besides being applicable only to single branches. In most regions of the Alps, district foresters estimate and report the extent of forest area affected by *C. rhododendri* to the responsible forest head office. The values are based on a visual estimate and do not include indications of the extent of symptom development, which can vary considerably between individual years, sites and specimens (Ganthaler *et al*., 2014; Zottele *et al*., 2014). In several physiological and biochemical studies, disease severity of individual trees was estimated via a semiquantitative method (Oberhuber *et al*., 1999; Ganthaler *et al*., 2017a); the percentage of current‐year needles with disease symptoms was evaluated by visual assessment and using a discrete disease scale with five classes between 0% and 100%. However, visual estimation is always subject to an individual's experience and reduces the repeatability (Bock *et al*., 2010). For analyses on seedlings or individual branches, this problem was solved by counting needles with and without disease symptoms to determine the exact percentage of needles affected (Ganthaler & Mayr, 2015). This time‐consuming and laborious approach was the only option to obtain an exact quantification of symptom development, but limited the number of samples and was not applicable to whole trees. Therefore, new methods with high reliability, practicability and a wider scale range are needed to improve disease quantification.

The development of a distinct yellow discolouration of infected spruce needles, characteristic for *C. rhododendri* and far more visible during mid‐summer, opens the possibility for disease quantification by image acquisition and analysis of foliar symptoms. The yellow needles (or yellow needle sectors) form a clear contrast to the green healthy needles and are easily detectable in the visible light spectrum and on true‐colour images. Simple red, green and blue (RGB) colour images have been used to detect biotic stress in several important crop plants such as wheat, cotton, bean, sugar beet, potato, tobacco, grapefruit and apple (Bock *et al*., 2008, 2010; Wijekoon *et al*., 2008; Camargo & Smith, 2009; Xie *et al*., 2012; Neumann *et al*., 2014; Stewart & McDonald, 2014; Sugiura *et al*., 2016) and very precise results have been obtained compared to visual assessment. Most of the comprehensive studies were conducted on imaging at leaf level, focusing on individual leaves with a simple wide leaf blade and providing the percentage of damaged leaf area. In addition, a high variety of image‐based methods has been developed for broad assessment of populations in the field, including hyperspectral, thermal as well as visible light imaging (Richardson *et al*., 2007; Mutka & Bart, 2015). This progress is widely based on the fast technical advancement of unmanned aerial vehicles (UAVs). The use of UAV‐based cameras can extend the survey area and provide flexible and timely monitoring with a high spatial ground resolution. This up‐and‐coming technology shows great potential for both agricultural surveillance and the detection of economically significant crop diseases (Di Gennaro *et al*., 2016; Sugiura *et al*., 2016), and is also gaining importance for forest monitoring. UAV‐based sensing techniques have been used, for example, to assess canopy cover, canopy height, gap patterns and species distribution in forests (Getzin *et al*., 2014; Chianucci *et al*., 2016; Zhang *et al*., 2016). This technology is particularly attractive for image acquisition of tree crowns and entire populations in hardly accessible dense forests, such as in alpine terrain, as well as to complement traditional field surveys. However, few studies combine image analysis on different spatial scales to match the multiscale structure of plants and diverse research approaches (Rousseau *et al*., 2015). Besides application in monitoring and management, image analysis can be an efficient tool for precise phenotyping in genetic and biochemical studies, to evaluate quantitative disease resistance on the plant or leaf level (Xie *et al*., 2012; Mahlein, 2016). Both image acquisition and image processing should be adjusted to the target of analysis (Mutka & Bart, 2015) and results need to be validated prior to application (Lobet, 2017).

The main aim of this study was to establish and validate a new method, applicable to individual branches and entire juvenile and adult trees, for the quantification of disease symptoms caused by *C. rhododendri*. Therefore, a procedure for image gaining, preprocessing and analysis was developed, including RGB colour photos acquired by a manual and a UAV‐based camera. Results obtained by image analysis were compared with values gained by manual counting of needles with and without disease symptoms and the utility, advantages and limits of the method are discussed. The study intended to provide a simple method, which can also be used by foresters, for a reliable diagnosis of rust disease in spruce forests.

## Materials and methods

### Study site

Image acquisition was conducted on the study site Praxmar im Sellraintal (Tyrol, Austria; 1680 m a.s.l., 47°09′N 11°08′E), located near the alpine timberline and characterized by typical subalpine Norway spruce forests, which were repeatedly infected and damaged in the last decade by *C. rhododendri*. Individual trees showing different disease severities and the occurrence of putative resistant trees (Mayr *et al*., 2001, 2010; Ganthaler *et al*., 2017a) offered ideal conditions for comparisons. Furthermore, 3‐year‐old plants from vegetative reproduction of spruce clones with varying susceptibility to *C. rhododendri* were available. All images were taken within 9 days (16 to 24 August 2016) during maximum symptom expression.

### Image recording

Images of individual branches and juvenile plants were taken with a standard digital camera (Coolpix AW130; Nikon CEE GmbH) in the automatic mode with a resolution of 4608 × 3456 pixels in jpeg format, and are subsequently referred to as ‘ground images’. Distance to the object was approximately 25 cm. This method included images of (i) individual branches of adult trees with natural background (*n *=* *20), (ii) cut branches with black background and constant light conditions (indirect daylight) in the laboratory (*n *=* *27), and (iii) juvenile plants growing in the field, in front of a black background (*n *=* *15). Individual branches of adult trees were up to 50 cm in length and included 5–18 apical shoots. Juvenile plants were 3‐year‐old cuttings and had a size of 10–15 cm. The black background was created with black velvet. Images of tree crowns were gained by using a semiprofessional remote‐controlled quadcopter (Phantom 2 Vision+; DJI) with a fixed remote‐controlled digital camera (resolution 4384 × 3288 pixels) in raw format (dng), and are subsequently referred to as ‘aerial images’. The Phantom 2 is a low‐cost high‐performance quadcopter ‘ready to fly’ with integrated camera, customized gimbal and a flight time of about 25 min per battery load. Flight altitude varied, depending on the tree height, between 7 and 15 m and the moveable gimbal enabled images with different aspects. The camera was operated in the automatic mode. This method included images of (iv) top views of individual crowns of adult trees (camera faced vertically downward during the flight; *n *=* *7), and (v) side views of tree crowns (camera faced horizontally; *n *=* *6). Each method included samples with a high variation in disease severity to test the precision and accuracy of the method.

### Image preprocessing and analysis

Preprocessing of both ground and aerial images was performed with the software photoshop lightroom v. 5 (Adobe Systems Inc.) and aimed at minimizing the influence of light conditions on the colour information, improving image quality and ensuring comparability and reproducibility of results. Steps included a standardized processing of the parameters white balance, blacks, exposure, clearness, contrast, saturation and colours. Detection of healthy and diseased plant parts was performed with the scientific software imageJ v. 1.45 (National Institutes of Health) on jpeg images composed of hue, saturation and brightness. Image sections of interest were selected manually with the function polygon selection. For individual branches and juvenile plants, selected areas covered the current‐year shoots, while for aerial images it was inevitable to include the entire crown. Pixels within this area, representing healthy needle parts or disease symptoms, were detected and measured automatically with the tools colour threshold and measure. The settings of the parameters for preprocessing and analysis (Table 1) were defined using three ground and three aerial images and then applied to all images. The disease severity was calculated based on the ratio of diseased needle area to total needle area.

**Table 1:** Settings for preprocessing and analysis of ground and aerial images for the detection of pixels representing needle parts of Norway spruce with and without symptoms of needle bladder rust

(a) Preprocessing with photoshop lightroom		
	Ground images	Aerial images
White balance	Adjusted with grey pixels	Adjusted with grey pixels
Blacks	−12	0
Exposure	0	−0.63
Clearness	+20	+20
Contrast	+9	+9
Saturation	+20	+20
Hue control	yellow +25, green +25	yellow +25, green +25
Hue saturation	yellow +50, green +50, orange +0, all others −100	yellow +50, green +50, orange +0, all others −100

(b) Analysis with imagej, tool colour threshold				
	Ground images		Aerial images	
	Diseased	Healthy	Diseased	Healthy
Saturation threshold	0–255	0–255	93–255	93–255
Brightness threshold	56–253	32–253	87–253	87–253
Hue threshold	27–48	49–101	27–44	45–101

### Determination of the disease severity by counting

For branches and juvenile plants previously photographed, the number of current‐year needles with and without rust symptoms was exactly counted and the percentage of diseased needles calculated. All needles with symptoms were counted as diseased, irrespective of a partial or complete discolouration. For tree crowns, one representative branch per tree was collected at breast height from the crown side selected for side view photographs and evaluated in the same manner. Selected branches showed a representative disease severity for the crown, as spruce individuals with equally distributed disease symptoms were chosen for analysis.

### Evaluation of the established method and statistics

To evaluate the precision, accuracy and reliability of the method, the severity of disease determined by image analysis (percentage needle area covered by disease symptoms) was correlated with the severity obtained by counting (percentage needles with disease symptoms). Linear regressions through the origin were calculated and the slopes (b), coefficients of determination (*R* ^2^) and levels of significance (*P*‐value) for the different approaches were compared. Analyses were carried out after testing for normal distribution (Kolmogorov–Smirnov test) and equality of variances (Levene's test). All tests were performed at a significance level of 5% using spss statistics 21 (IBM).

## Results

### Required image quality and software settings

**Figure 1:**
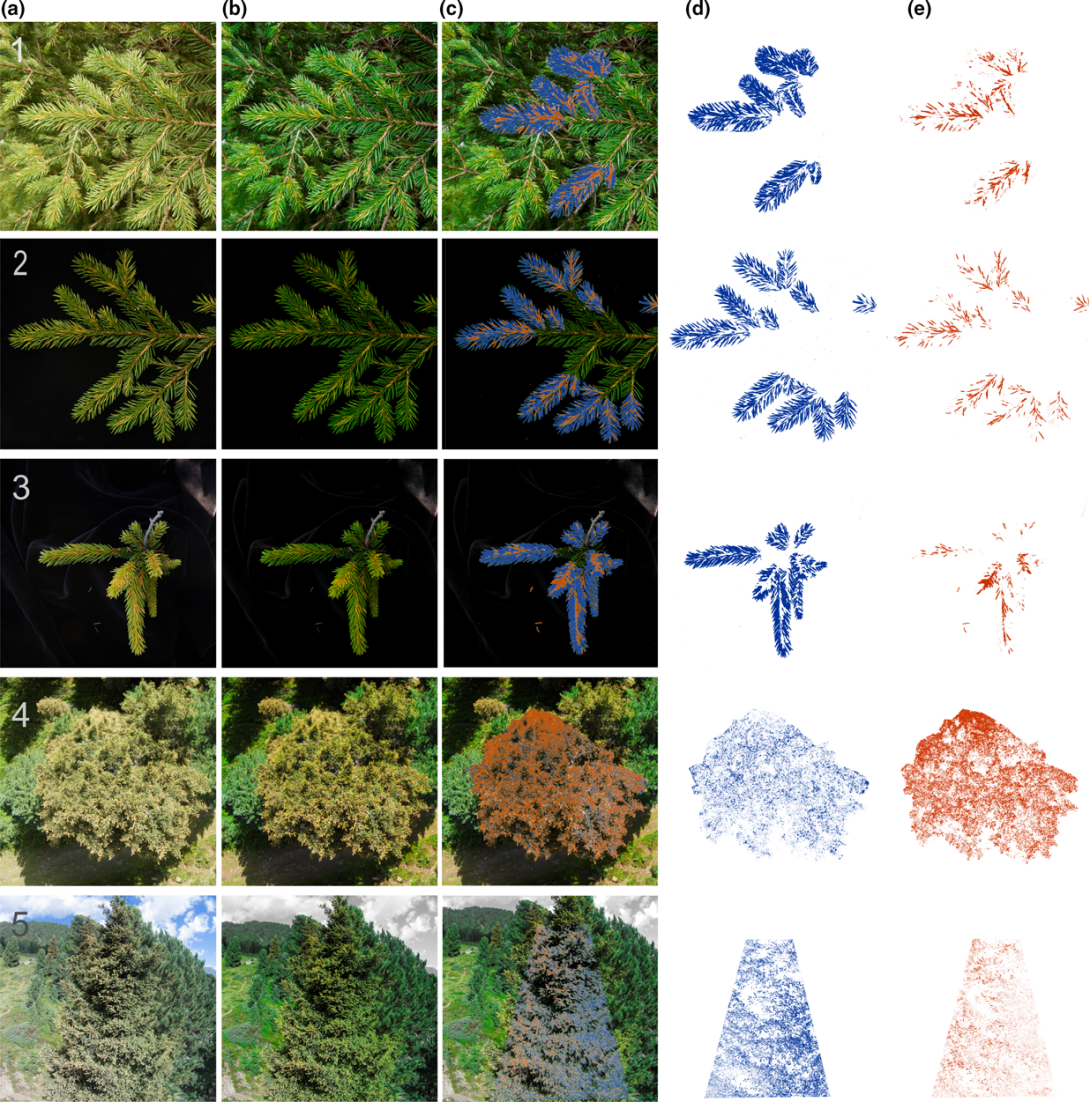
Image processing workflow shown for ground (1–3) and aerial (4–5) images of Norway spruce trees infected by *Chrysomyxa rhododendri*: (a) original image, (b) preprocessed image, (c) selection of disease symptoms (in red) and healthy plant parts (in blue), (d) determined area covered by healthy needle tissue and (e) area covered by disease symptoms.

**Figure 2:**
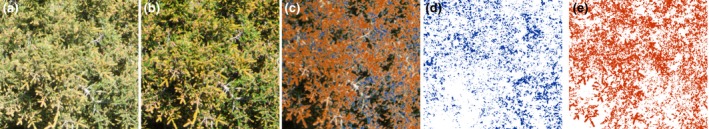
The image processing workflow shown for a detail of an aerial image (top view of the crown) of Norway spruce trees infected by *Chrysomyxa rhododendri*: (a) original image, (b) preprocessed image, (c) selection of disease symptoms (in red) and healthy plant parts (in blue), (d) determined area covered by healthy needle tissue and (e) area covered by disease symptoms.. Disease symptoms and healthy plant parts were accurately recognized, dark areas and branches were excluded.

**Figure 3:**
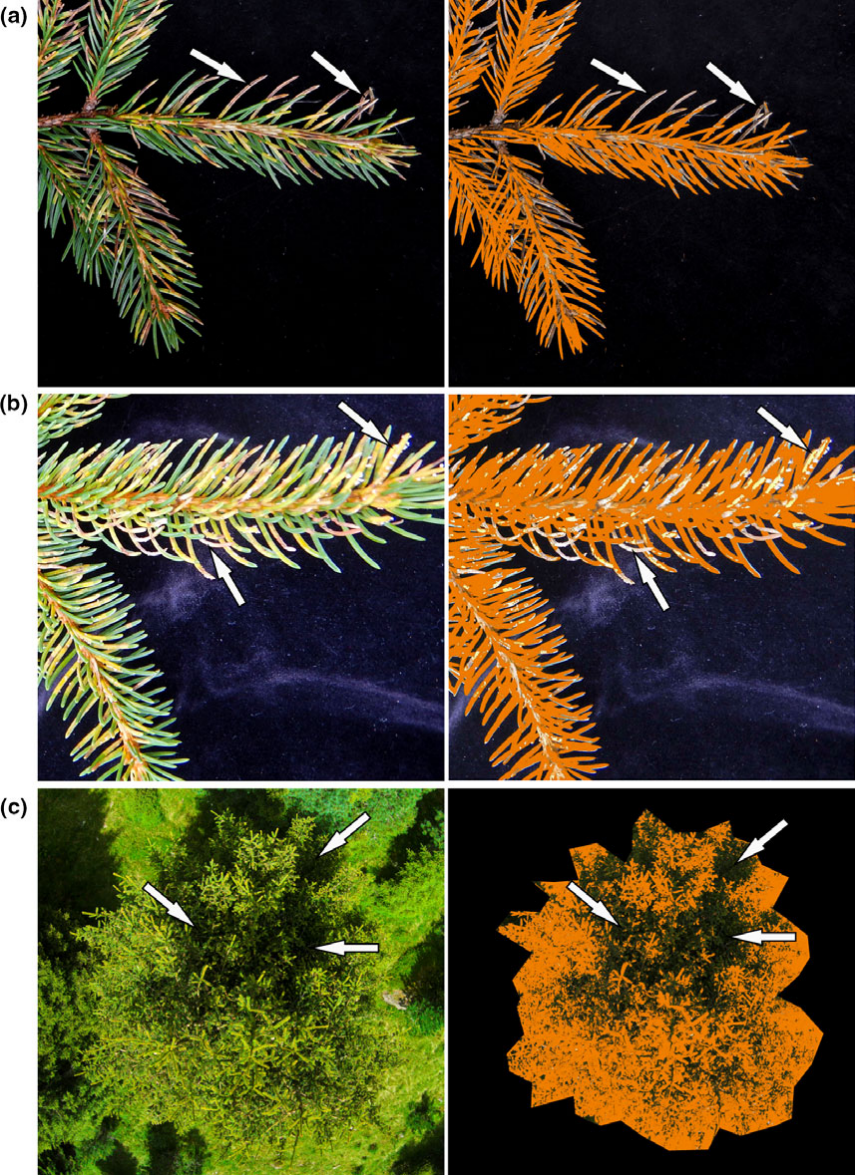
Different sources of error caused by wrong time of image acquisition or poor image quality. The original images and the processed images are shown (left hand and right hand columns, respectively); areas with appropriate colour information are marked in orange, while areas inappropriate for analysis are unmarked and indicated by arrows. (a) Brownish needles occurring during the advanced stage of the disease are not detected properly because they are outside the colour thresholds set for disease symptoms, (b) light reflectance and overexposure cause gaps with missing colour information, and (c) large shaded areas are too dark to extract colour information. The photographs shown represent extreme examples of inappropriate images and were not included in the analysis.

The presented protocol enabled a distinct selection of needle parts with and without disease symptoms caused by *C. rhododendri* on ground and aerial photographs (Figs 1 & 2). Critical evaluation of selected image areas revealed several important preconditions regarding image quality. Direct solar radiation causing light reflectance on the needle surface could reduce the colour information of images and cause gaps in the analysis (Fig. 3b). The same applied to shaded areas within tree crowns (Fig. 3c). Moreover, images that were acquired after infected needles started to decompose and become brownish were not suitable (Fig. 3a). Optimal software settings during image preprocessing and analysis (including thresholds of the channels saturation, brightness and hue; Table 1) for best detection of pixels showing healthy and diseased plant parts varied slightly between ground and aerial images. Stricter saturation and brightness thresholds for aerial images were necessary to prevent incorrect evaluation of shaded and overexposed areas.

### Disease severity extracted from ground images

**Figure 4:**
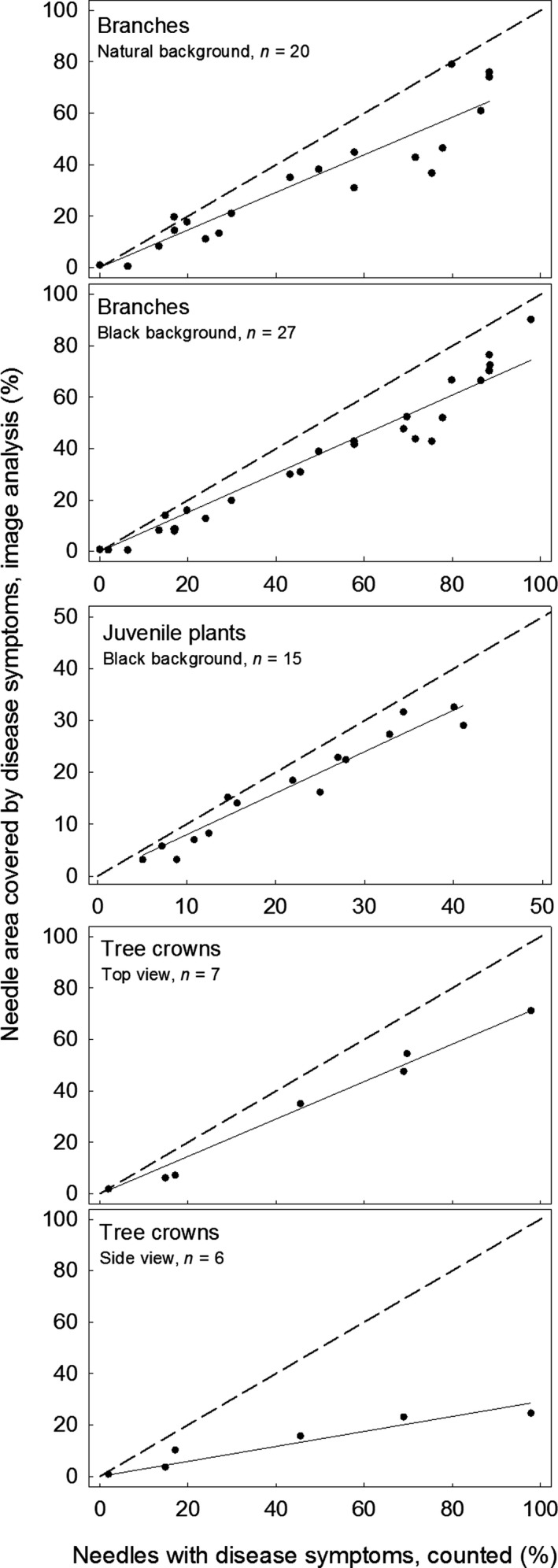
Correlations of the disease severity determined by image analysis (% of needle area with disease symptoms) and by counting (% of needles with disease symptoms) for the different types of images used. The dashed line indicates the concordance line and the continuous line the linear regression between the two assessment methods.

Analysis of ground images following the established protocol resulted in disease severities that were highly correlated with the values obtained by manual counting (Fig. 4). The percentage of needle area covered with disease symptoms obtained by image analysis was lower in 73 out of 75 observations than the percentage of diseased needles obtained by counting. Precision (coefficient of determination) and accuracy (slope of the linear relationship) were highest for branches photographed with black background (images made under constant light conditions in the laboratory; *R* ^2^ = 0.95, b = 0.76) and juvenile plants photographed with black background (under field light conditions; *R* ^2^ = 0.94, b = 0.80), and were lowest for branches with natural background (*R* ^2^ = 0.87, b = 0.73; Table 2).

**Table 2:** Characteristics of the linear regression between disease severities determined by counting and by image analysis

	b	*R* ^2^	*P‐*value
Branches (natural background)	0.73 ± 0.04	0.87	<0.001
Branches (black background)	0.76 ± 0.02	0.95	<0.001
Juvenile plants (black background)	0.80 ± 0.03	0.94	<0.001
Tree crown (top view)	0.73 ± 0.02	0.98	<0.001
Tree crown (side view)	0.29 ± 0.03	0.88	0.003

b, slope; *R* ^2^, coefficient of determination; *P*, level of significance of the regression.

### Disease severity extracted from aerial images

Similarly to ground images, analysis of aerial images of tree crowns revealed results to be highly correlated with the number of needles with disease symptoms counted on a branch at breast height. For images showing the crown from the top, the precision and accuracy were similar to ground images (*R* ^2^ = 0.98, b = 0.73), while side view images resulted in significantly lower disease severity estimates (b = 0.29) combined with a lower coefficient of determination (*R* ^2^ = 0.88). Needle areas with and without disease symptoms were accurately recognized and distinguished from woody stem sections, which remained unselected (Fig. 2c).

## Discussion

The study demonstrated that a well‐defined signal related to needle bladder rust symptoms of Norway spruce can be extracted from images recorded by standard digital cameras and using semiprofessional lightweight drones, and that images in the visible range can be effectively used for disease quantification. Despite the relatively simple, time‐ and cost‐efficient image taking and processing method, the precision of the linear relationship between the result obtained by manual counting and the new image analysis method is comparable to results in other studies on disease detection from true colour images. This includes quantification of necrotic leaf areas caused by citrus canker on grapefruit leaves (*R* ^2^ = 0.77–0.83, Bock *et al*., 2008), common bacterial blight symptoms on bean leaves (*R* ^2^ = 0.87–0.94, Xie *et al*., 2012), and number of pycnidia formed by *Zymoseptoria tritici* on wheat (Lin's concordance correlation coefficient = 0.93, Stewart & McDonald, 2014). Measurements made at short distance provided higher resolution and allowed for easier illumination control, but displayed limited areas. In contrast, the use of UAVs and aerial‐based sensing enabled a wider range of analysis and potential points of view than ground surveys. The combination of both techniques thus could broaden the application options and provide the right approach for different needs.

The values gained with the presented method reflected the needle area with distinct symptoms, while counting determined the number of affected spruce needles regardless of the extent of disease symptoms. This may explain the discrepancy between needle counting and image analysis (slope of the linear relationship, b < 0.8) and may contribute to the deviation of individual values from linear regressions. However, the needle area with distinct symptoms may be a better reference value for physiological analyses like measurements of photosynthesis, biomass production, secondary compound accumulation and pathogen defence reactions (Oberhuber *et al*., 1999; Plattner *et al*., 1999; Ganthaler *et al*., 2017b), and may be more representative for estimations of the quantitative resistance of trees against needle bladder rust. Moreover, the determined slope of the linear regressions permits the comparison and conversion of values gained with one of the two methods.

The pathogen *C. rhododendri* infects only the young, sprouting spruce needles (De Bary, 1879; Gäumann, 1946) and therefore, the determined disease severity should refer to healthy and diseased needles of the current‐year foliage. Older needles, which could potentially cause an underestimation of disease severity if included in the analysis, can easily be excluded in images of individual juvenile plants and branches during the image processing. For aerial images, current‐year needles are well represented if the crown is photographed from the top, because they hide older needles. It should be considered that cone‐shaped trees allow imaging of the entire crown, while images of columnar trees display only the upper part of the crown. Side view images of trees, however, depicted older needles as well as current‐year needles because of the branching pattern and caused significant underestimations and higher variance of the disease severity compared to top views and ground images. Additional variance can be attributed to several factors: a partial overlap of needle areas on the two‐dimensional photos, masking of diseased needles by healthy needles, shading of needles by asymmetric branching patterns and wrongly detected pixels in the background. These limitations will need to be overcome and results need to be proved by using a bigger sample number to enable precise quantification while using side view images. Comparison of drone images with the count of one representative branch may also have caused additional variance, although trees with homogeneous disease manifestation were carefully selected. Images with poor quality or brownish needles represent a source of error and should be excluded from analysis. It is recommended that image acquisition is conducted in the second half of August on cloudy days around midday to avoid brownish needles, light reflectance and large shadows, and to ensure the consistency of long‐term monitoring. An additional grey scale calibration board placed on the images could be used to improve the white balance of images taken under different light and weather conditions. Attention should be paid to prevent artefacts or flaws due to discolorations caused by the physiological condition of analysed trees or additional biotic and abiotic stress (Bock *et al*., 2010).

The presented procedure for quantification of disease symptoms caused by *C. rhododendri* is precise and objective, and at the same time efficient and applicable to single branches as well as entire trees. This combination of features is not attainable with traditional methods, as they are either precise and objective but time‐intensive and limited to single branches (manual counting of diseased needles) or time‐efficient and applicable to entire trees but dependent on the rater's experience (visual assessment). The estimated time needed to evaluate individual branches using the image analysis method was less than 20% of the time needed for manual counting. For aerial images a relatively inexpensive quadcopter (current price of the Phantom 2 including camera <€600) was used, which was easy to operate after short training, lightweight (1.3 kg), could be transported in a backpack and enabled easy replacement of damaged components. The average time required was approximately 3–10 minutes per aerial image, depending on the distance of analysed trees and the number of take‐offs needed. The use of RGB cameras reduced the equipment costs considerably compared to approaches with multispectral and thermal cameras. Moreover, the defined image processing protocol enabled a semi‐automated, quick and impartial detection of pixels representing disease symptoms, with a high precision throughout the different approaches and tested scale levels.

The new technique for estimation of *C. rhododendri* disease severity in the field is likely to be useful for both practical and scientific application. In forest cultivation and management, the presented method could offer a novel opportunity to implement a monitoring programme and stimulate the investigation of the spatial and year‐specific variation in disease severity. The establishment of long‐term records for individual trees by repeated UAV flights or the evaluation of branch and juvenile plant images with respective GPS coordinates sent by foresters could contribute to a better localization of heavily affected Norway spruce populations. Based on this information, foresters could develop adapted afforestation strategies and recommend planting of other tree species (e.g. *Pinus cembra)* instead of *P. abies* in particular areas. In addition, UAV flights could facilitate the identification and GPS localization of trees with enhanced resistance for breeding programmes (Ganthaler *et al*., 2014, 2017a), which would be cheaper than using helicopter flights. Application on entire forest stands and larger regions would require more cost‐intensive professional UAVs, georeferencing and alignment of images (Getzin *et al*., 2014; Di Gennaro *et al*., 2016; Haghighattalab *et al*., 2016). However, a more precise and comprehensive determination of the quantitative variation in disease severity and susceptibility to infection strongly benefits genetic, biochemical and physiological studies, as improved phenotyping is a current challenge to meet the increasing high‐throughput capacity of genotyping (Houle *et al*., 2010). Image analysis can enhance the ability to distinguish between genotypes with different levels of susceptibility and is highly useful for investigating quantitative genetics of disease resistance (Xie *et al*., 2012). Moreover, imaging techniques represent an innovative methodology to analyse the distribution of disease symptoms within forest stands and individual crowns, as well as potential links with topography, orientation, slope and climate parameters (Ganthaler & Mayr, 2015).

In summary, this study demonstrates true‐colour imagery to be an efficient analysis tool for detection of tree crown damage caused by *C. rhododendri*. It underlines the great potential for the application of aerial image analysis in forest ecosystems, which requires improvements similar to those recently achieved in agriculture. Results indicate that the image processing protocol developed could replace the conventional assessment of damage caused by needle bladder rust in spruce forests. Furthermore, it might be easily adapted to other biotic or abiotic diseases of forest trees causing characteristic discolouration patterns.
